# Parent-Reported Satisfaction, Perceived Child Acceptance, and Ease of Administration of a Strawberry-Flavoured Ibuprofen Suspension for Children: A Cross-Sectional Survey of Experienced Users in Germany

**DOI:** 10.3390/jmahp14030056

**Published:** 2026-09-18

**Authors:** Kerstin Weber, Spasenija Bradic, Gábor Kertes, Marcel Konrad, Karel Kostev

**Affiliations:** 1 Paediatrics and Adolescent Medicine, 12159, Berlin, Germany; 2 Reckitt Benckiser Deutschland GmbH, 69115 Heidelberg, Germany; 3 Health & Social, FOM University of Applied Sciences for Economics and Management, 60486 Frankfurt am Main, Germany; 4 University Hospital, Marburg University, 35043 Marburg, Germany

**Keywords:** paediatric analgesia, ibuprofen, medication adherence, palatability, perceived child acceptance, oral suspension, parental survey, market research, parent-reported outcomes, Germany

## Abstract

Background: Palatability and ease of administration are recognised as key determinants of medication adherence in paediatric populations. Oral ibuprofen suspensions are among the most commonly used analgesic and antipyretic medications for children in Germany, yet parental and parent-perceived child perspectives on these formulation attributes remain underreported for the German market. Methods: A cross-sectional, online market-research survey, commissioned to substantiate potential product communication claims, was conducted among 1005 parents residing in Germany, each of whom had administered a strawberry-flavoured ibuprofen suspension (Nurofen Junior Fever and Pain Suspension Strawberry, Reckitt Benckiser) to at least one child aged 3 months to 12 years within the preceding 12 months. Closed-ended items assessed overall satisfaction, willingness to recommend, parent-perceived child acceptance, perceived ease of administration, and taste satisfaction; all product-specific statements (Q17 and Q18) used a binary agree/do-not-agree format without a neutral option. Results: Overall satisfaction with the product was reported by 99% of respondents (995/1005), and 98% (985/1005) stated they would recommend it to other parents. Agreement rates exceeded 90% for all primary claim statements (Q18): easy and safe to administer (96%), satisfied with how well the child takes the medicine (92%), well accepted by child (92%), satisfied with flavour (92%), good feeling child takes medicine without problems (91%), child finds taste pleasant (90%), child likes the taste (90%), and child takes it without problems (88%). For comparative acceptance statements (Q17), agreement ranged from 83% to 93%. Fever was the primary indication for use (91%), and the product was used a mean of 3.3 times per year. Conclusions: These findings provide consumer-reported, real-world survey data indicating that, among surveyed parents with recent personal experience of using the product, reported satisfaction and perceived child acceptance were high. Because participation required experience with the product within the previous 12 months, while allowing concomitant experience with other paediatric products, and because fever and pain episodes commonly occur more than once over a year in children, the study relied on parent-reported outcomes. The study was designed as a descriptive survey of user experiences and was not intended as a comparative evaluation; accordingly, no comparator was included. The findings should therefore be interpreted as descriptive of this study population and cannot be used to infer comparative performance, improved adherence, or better clinical outcomes; they may, however, help identify taste and ease of administration as candidate domains of paediatric acceptability to be examined in future, more rigorously designed studies.

## 1. Introduction

Adequate management of fever and pain in children represents one of the most frequent clinical challenges in primary care and family medicine. Oral ibuprofen suspensions are a cornerstone of paediatric analgesia and antipyresis across Europe and constitute a substantial proportion of over-the-counter medication use in the paediatric population. In Germany, mild to moderate pain and fever in children aged 3 months to 12 years are among the leading indications for parental self-medication, with ibuprofen suspensions routinely purchased without prescription. A recent real-world analysis of prescribing patterns across 250 German paediatric practices confirmed that ibuprofen is the dominant agent for acute pain management in children aged 2–12 years, accounting for 75% of all prescriptions, with oral suspension being the most frequently prescribed dosage form, particularly in younger children aged 2–5 years (68.2%) [1].

Beyond pharmacological efficacy, the success of oral paediatric medications depends critically on formulation acceptability—a concept encompassing palatability (taste, smell, texture, and aftertaste), ease of administration, and the ability and willingness of both child and caregiver to use the product as intended. The European Medicines Agency (EMA) addressed this early in its Reflection Paper on Formulations of Choice for the Paediatric Population, which explicitly highlighted taste and palatability as determinants of therapy adherence in children [2]. Children are not merely small adults: developmental differences in taste perception, a documented preference for sweet flavours, and a heightened sensitivity to bitter stimuli render palatability a primary, not secondary, consideration in formulation design [3,4].

The consequences of poor palatability extend well beyond a single refused dose. Incomplete or inconsistent medication intake—often driven by taste aversion—undermines therapeutic outcomes, increases the risk of treatment failure, and imposes additional burdens on caregivers [5,6]. Winnick et al. (2005) demonstrated that palatability is one of the strongest modifiable predictors of medication compliance in children [7]. More recently, a cross-sectional survey among general practitioners and pharmacists in Ireland found that 76.9% of GPs reported deviating from prescribing guidelines specifically to improve child adherence, primarily due to palatability concerns [8]. The systematic challenge of poor-tasting paediatric medicines is further documented in a comprehensive scoping review identifying palatability as a barrier to adherence across 77 disease areas and 156 medicinal products [9].

Strawberry flavouring has emerged as a particularly well-accepted taste profile for oral paediatric medications in European studies. Thompson et al. (2013) reported that 85.3% of children aged 6–12 years rated a strawberry-flavoured lozenge as ‘good’, ‘really good’, or ‘super good’ on a hedonic facial scale, significantly outperforming an orange-flavoured equivalent (49.0%; *p* < 0.0001) [10]. A German hospital study on analgesic and antipyretic formulations similarly found that ibuprofen in a strawberry-flavoured oral suspension, delivered with an oral syringe, was positively accepted, whereas an orange-flavoured paracetamol presented with a measuring cup was not [11]. These findings underline that both flavour choice and administration device meaningfully affect child acceptance.

A consideration specific to the German market is how these products are dispensed once selected. Under the statutory aut-idem rule (§129 of the German Social Code Book V, SGB V) and the associated discount agreements (Rabattverträge) negotiated between statutory health insurers and manufacturers, pharmacists are generally obliged to substitute a prescribed medicine with the most cost-effective product containing the same active ingredient, unless the prescriber explicitly excludes substitution [12]. While indispensable for cost containment, this mechanism implicitly assumes that products sharing an active ingredient are interchangeable in practice. For paediatric oral liquids that assumption is complicated by formulation acceptability: a product that a child refuses, or takes only partially, may not deliver the intended therapeutic exposure regardless of its lower acquisition price. Whether child acceptability should therefore be weighed alongside price in substitution and dispensing decisions is an open policy question that the present descriptive survey cannot resolve, but to which it can contribute preliminary real-world acceptability data. The present survey was designed to help address this evidence gap by quantifying parental and parent-perceived child acceptance of a widely used strawberry-flavoured ibuprofen suspension under everyday conditions of use.

Despite growing evidence on palatability as a driver of adherence, large-scale, real-world parental surveys specifically addressing an ibuprofen suspension in the German paediatric market are lacking. Most published data derive from clinical taste-testing studies in controlled settings, which may not fully reflect the everyday caregiver experience. The present study addresses this gap by reporting results from a cross-sectional survey of 1005 German parents who had actual, recent experience administering a strawberry-flavoured ibuprofen suspension to their children. The primary objective was to characterise parent-reported taste acceptance, parent-perceived child acceptance, and ease of administration of a strawberry-flavoured ibuprofen suspension under real-world conditions of use. Secondary objectives included characterising category purchase drivers and usage patterns in the German paediatric analgesic/antipyretic market. Framed by the German substitution and dispensing context outlined above, a further, exploratory objective was to generate preliminary, hypothesis-forming acceptability data relevant to the broader question of whether child-centred acceptability might be considered alongside price when paediatric analgesic/antipyretic products are prescribed, dispensed, or substituted—a question this descriptive survey can inform but not answer.

## 2. Materials and Methods

### 2.1. Study Design

This was a cross-sectional, online quantitative survey conducted in Germany in May 2026. The study was commissioned by Reckitt Benckiser GmbH and implemented by The Lifesights Company GmbH (Bremen, Germany). The survey adhered to the ICC/ESOMAR International Code on Market, Opinion and Social Research. Questionnaire development, data collection, response coding, and statistical analysis were carried out independently by The Lifesights Company GmbH. The sponsor (Reckitt Benckiser, Heidelberg, Germany) defined the survey objectives and reviewed the final research report but did not take part in data collection, response coding, or the calculation of the results reported here. Parents provided feedback through a claim-substantiation survey; the product-specific statements were developed by The Lifesights Company to test potential claims and did not constitute a formally validated caregiver-reported outcome instrument. No psychometric validation, concept elicitation, or cognitive interviewing was undertaken.

The sponsor did not hold veto rights over the present manuscript.

### 2.2. Study Population and Eligibility

Eligible participants were adults aged 18 years or older, residing in Germany. All respondents had to be a parent or legal guardian of at least one child aged between 3 months and 12 years living in the household, and at least equally responsible—compared to a partner or others—for both purchasing and administering pain relief medication to their child or children. In addition, all participants were required to be aware of Nurofen for Children (Nurofen Junior Fever and Pain Suspension) and to have personally administered the strawberry-flavoured variety to at least one eligible child within the preceding 12 months. Participants were excluded if employed in a sensitive industry (e.g., healthcare, market research, advertising, or pharmaceuticals) or if they had participated in market research within the preceding three months. Eligibility was not restricted to brand-exclusive users: parents were also aware of and, in many cases, had previously given their child other paediatric pain and fever syrup brands. 85% of parents had at some point given their child a brand other than Nurofen Junior Fever and Pain Suspension. Where a respondent had more than one eligible child, the questions referred to the child to whom the parent had most recently given the strawberry-flavoured product (the index child); the number of children aged 3 months to 12 years in the household was recorded as a profiling item. The lower eligibility bound of 3 months corresponds to the German marketing authorisation for the study formulation (Nurofen Junior Fever and Pain Suspension Strawberry, ibuprofen 40 mg/mL), which is indicated from 3 months of age and a body weight of at least 5 kg [13].

### 2.3. Sample Size and Recruitment

A total of 1005 participants were recruited through an online consumer panel. Sample size was determined to allow robust estimation of proportions at the 95% confidence interval level with a margin of error of approximately ±3 percentage points under the conservative assumption of an expected proportion of *p* = 0.5 (maximum variance); for the substantially higher proportions actually observed, the corresponding margins are narrower, as reflected in the Wilson confidence intervals reported below. No power calculation for hypothesis testing was performed, consistent with the descriptive and substantiation purpose of the survey. Respondents were recruited using quota-based sampling to ensure a gender distribution reflective of the German parent population. Beyond gender, no quotas were applied for region, education, income, employment, household composition, or urban/rural residence, and these characteristics were not collected; the representativeness of the sample beyond parent gender therefore cannot be established (see Limitations Section). Participants received a modest, non-coercive incentive for taking part, provided in the form of redeemable panel points, consistent with standard access-panel practice; the incentive was not designed to influence the answers given. The panel provider applied data-quality controls throughout fieldwork, including verification of panellist identity and prevention of duplicate participation (deduplication by panellist ID, device, and IP address), speed checks to detect and remove unrealistically fast completions, attention and logical-consistency checks (including trap/plausibility questions), detection of straight-lining and other low-quality response patterns, and fraud- and bot-detection measures; respondents failing these checks were excluded before quotas were enforced on the cleaned, qualified sample of 1005.

### 2.4. Survey Instrument

The questionnaire was administered in German and covered four thematic modules: (1) category understanding, encompassing purchase decision criteria, usage occasions, administration frequency, and formulation formats used alongside syrup; (2) experience with and statement agreement for the study product; (3) brand awareness and usage within the paediatric analgesic/antipyretic syrup category; and (4) profiling questions. The present report focuses on Modules 1 and 2, which are directly relevant to the primary and secondary study objectives. Profiling items additionally recorded parent gender and age and the number of children aged 3 months to 12 years in the household; of these, parent gender and child age are reported here, as no further demographic breakdowns were available in the research dataset.

Agreement with product-specific statements was assessed using a binary agree/do not agree format across two question blocks (Q17 and Q18). Satisfaction and recommendation items used yes/no response formats. Purchase decision criteria (Q19, Q20) were rated on a 5-point Likert scale (1 = not important at all; 5 = very important); proportions are reported as Top-2 Box scores (important + very important). A binary agree/do-not-agree format was chosen deliberately for the acceptance and experience claims (Q17 and Q18): these items were framed as clear, decision-relevant endorsements that respondents could answer quickly and unambiguously across a large number of statements, reducing respondent fatigue and the interpretive ambiguity that intermediate response categories can introduce for evaluative statements of this kind. In contrast, the purchase-decision criteria (Q19 and Q20) required a graded judgement of relative importance, for which a 5-point Likert scale is the established format, because the analytic aim there was to rank the relative weight of many competing criteria rather than to record a simple endorsement. We acknowledge that this design trades strength-of-agreement granularity for breadth and clarity, and that binary endorsement items are susceptible to ceiling effects (the format offered no neutral or ‘don’t know’ option, so ‘do not agree’ pools disagreement, uncertainty, and indifference, and the positively framed, claim-substantiation wording may raise agreement through acquiescence and positive-framing bias); this is addressed in Limitations Section.

### 2.5. Statistical Analysis

Data were analysed descriptively. Frequencies and proportions are reported for all categorical items. Subgroup analyses by age of child (3 months to 5 years vs. 6 to 12 years) are reported for agreement statement items. All analyses were performed on the total sample unless otherwise stated. Missing data were handled by calculating percentages based on valid responses to each item. To reconcile the reported ±3-percentage-point precision with the point estimates, Wilson score 95% confidence intervals (CIs) were calculated for the key proportions. Differences between the two age subgroups were tested using the two-proportion z (χ^2^) test, with a two-sided *p* < 0.05 regarded as statistically significant. These inferential statistics are intended as descriptive precision estimates rather than confirmatory hypothesis tests, consistent with the substantiation purpose of the survey. Because a large number of subgroup comparisons were performed across Q17 and Q18 and the survey was descriptive in purpose, these subgroup tests are regarded as exploratory; no adjustment for multiple comparisons was applied, and individual *p*-values are interpreted with caution and reported alongside absolute percentage-point differences rather than as confirmatory findings. Given the large sample, small absolute differences can reach statistical significance without necessarily being practically meaningful. The study is reported in accordance with the STROBE guideline for cross-sectional studies, adapted for a market-research survey, and the completed checklist is provided as Supplementary Material [14].

### 2.6. Ethical Considerations

The study was conducted as a non-interventional market-research survey under the ICC/ESOMAR International Code on Market, Opinion and Social Research. Three distinct requirements should be distinguished. (i) Data protection: no directly identifiable personal data were collected or retained, and data were processed in anonymised form, so that under the EU General Data Protection Regulation (Recital 26) and the German Federal Data Protection Act (BDSG) the processing did not concern identifiable individuals. (ii) Informed consent: all participants were informed of the survey’s purpose and of its voluntary and anonymous nature and gave digital consent to take part before commencing; this was consent to participate in an anonymous survey, not consent for the processing of identifiable health data, of which none were collected. (iii) Research-ethics review: as a non-interventional, anonymous market-research survey that performed no medical procedures, the study did not fall under the statutory remit requiring approval by a medical ethics committee in Germany, and no formal ethics-committee vote or exemption determination was obtained, and none was sought, consistent with the study protocol’s classification of this market-research survey as falling outside the scope of studies requiring ethics-committee review. Data-protection exemption under the GDPR is separate from research-ethics review.

## 3. Results

### 3.1. Sample Characteristics

The achieved sample and the age distribution of the index child are summarised in Table 1. The sample comprised 70% women and 30% men, and the age of the child most recently given the study product spanned the full eligible range of 3 months to 12 years. Only parent gender and child age were available as sample characteristics; no data on geographic region, education, income, employment, household composition, child sex, or urban/rural residence were collected, which limits assessment of the sample’s representativeness of the German parent population (see Limitations Section).

### 3.2. Overall Satisfaction and Willingness to Recommend

Overall satisfaction with the strawberry-flavoured ibuprofen suspension was reported by 99% of respondents (995/1005). Willingness to recommend the product to other parents was confirmed by 98% of participants (985/1005). Both rates were consistent across the two age subgroups: satisfaction was 97% among parents of children aged 3 months to 5 years (n = 428) and 99% among parents of children aged 6 to 12 years (n = 577); willingness to recommend was 96% and 99% in the respective subgroups. Wilson 95% CIs around these estimates were narrow, reflecting the large sample: overall satisfaction 99% (95% CI 98–99%) and willingness to recommend 98% (95% CI 97–99%). The higher rates among parents of children aged 6–12 years were statistically significant but small in absolute terms for both satisfaction (99% vs. 97%; 2-percentage-point difference; *p* = 0.02) and willingness to recommend (99% vs. 96%; 3-percentage-point difference; *p* = 0.002); as exploratory comparisons, these should not be over-interpreted.

### 3.3. Agreement with Comparative Acceptance and Taste Statements (Q17)

Table 2 presents agreement rates for six comparative statements about Nurofen Junior Fever and Pain Suspension Strawberry relative to other medicines and in the context of administration experience. Because respondents were not randomised to competing products and their prior exposure to alternatives was heterogeneous, these comparative items reflect subjective, retrospective parental impressions rather than head-to-head comparative evidence. All six statements achieved agreement levels above 80% in the total sample. The highest agreement was observed for the statement that the product gives fewer administration struggles (93%), followed by high child acceptance (90%). Statements pertaining directly to taste preference—that the product has the taste most preferred by the child (88%) and that it ranked highest for most preferred taste (87%)—achieved agreement levels above 85%, consistent with the perceived importance of flavour in parent-reported child acceptance. Agreement that the product is better accepted than other medicines in syrup form was also 87%, and that it is the medicine the child is most willing to take reached 83%. Agreement was consistently higher in the 6 to 12 year age group than among parents of younger children, though the pattern was uniformly positive across both subgroups. Wilson 95% CIs for the total-sample proportions spanned approximately ±2–3 percentage points (for example, 93% [95% CI 91–94%] for fewer administration struggles and 83% [95% CI 81–85%] for the medicine the child is most willing to take). In these exploratory comparisons (no correction for multiple testing), age-subgroup differences were statistically significant for the taste-related statements—taste most preferred by the child (90% vs. 84%; *p* = 0.006), number-one product for most preferred taste (89% vs. 84%; *p* = 0.02), and better accepted than other syrups (90% vs. 83%; *p* = 0.001)—but not for fewer struggles (*p* = 0.54), high acceptance (*p* = 0.63), or most willing to take (*p* = 0.22); these differences of 5–7 percentage points are interpreted cautiously. The direction of the age difference was nevertheless consistent across items, favouring the older age group throughout; possible explanations for this pattern, and the distinction between statistical significance and practical relevance, are considered in Section 4.

### 3.4. Agreement with Product-Specific Experience Statements (Q18)

Table 3 presents agreement rates for eight statements drawn from direct parental experience with the product (all parent-reported). All eight statements achieved agreement levels of at least 88% in the total sample. The highest agreement was observed for ease and safety of administration (96%). Satisfaction with how well the child takes the medicine (92%), child acceptance (92%), and satisfaction with the flavour (92%) ranked equally, followed by the feeling of confidence that the child takes the medicine without problems (91%). Two taste-specific parent-perceived statements achieved 90% agreement each: that the child finds the taste pleasant and that the child likes the taste. The statement that the child takes the product without problems—without fussing or spitting out—reached 88%. Agreement was consistently higher in the 6 to 12 year age group, most notably for the item on satisfaction with how well the child takes the medicine (89% vs. 95%) and for the child liking the taste (86% vs. 93%). Total-sample Wilson 95% CIs ranged from 95–97% (ease and safety of administration, 96%) to 86–90% (takes the product without problems, 88%). In exploratory age-subgroup comparisons (no adjustment for multiple testing), the higher agreement among parents of older children was statistically significant for satisfaction with how well the child takes the medicine (95% vs. 89%; *p* < 0.001), the child liking the taste (93% vs. 86%; *p* < 0.001), satisfaction with the flavour (94% vs. 89%; *p* = 0.005), the good-feeling item (93% vs. 88%; *p* = 0.007), the child finding the taste pleasant (92% vs. 88%; *p* = 0.04), and the child taking the product without problems (90% vs. 85%; *p* = 0.02); it was not significant for ease and safety of administration (*p* = 0.48) or overall child acceptance (*p* = 0.08). These absolute differences (4–7 percentage points) are small and are not interpreted as evidence of practically meaningful differences in acceptability. They were, however, consistently in the same direction, and for several items the Wilson 95% confidence intervals of the two age groups did not overlap; this age pattern and its possible explanations are discussed in Section 4.

### 3.5. Purchase Decision Criteria (Q19 and Q20)

Table 4 presents the full ranking of product attribute criteria for the purchase decision. Tolerability (96%), ease of administration (95%), fast-acting effect (95%), ease of dosing (93%), and long-lasting effect (92%) were rated as important or very important by more than nine in ten respondents. The criterion that the child does not refuse or make a fuss was rated important by 91% of parents, ranking sixth overall and immediately before the criterion that the product has a taste the child likes (87%). This indicates that, in parents’ self-reported ratings, child acceptance and taste were among the more highly rated purchase criteria, ranked below tolerability, efficacy, and administration parameters; as stated-importance ratings, they reflect perceived importance rather than demonstrated determinants of actual purchasing behaviour. A dosage syringe being available alongside the product was rated important by 83% of parents, jointly with the criterion of strong effect. Absence of artificial additives was cited by 59%, while ingredient-specific preferences (ibuprofen content, sugar-free, allergen-free, paracetamol content or absence) attracted ratings between 16% and 48%.

Table 5 presents the importance ratings for brand reputation criteria. Brand trust was the leading reputational driver (73%), followed by paediatrician recommendation (71%). Brand identity as a pain relief expert (64%) and general brand recognition (62%) ranked third and fourth. Pharmacy staff recommendation (59%), price (47%), and recommendation by friends or acquaintances (42%) were rated lower, indicating that professional endorsement and brand credibility outweigh social and economic factors in this category.

### 3.6. Usage Occasions, Frequency, and Administration Context

Fever was by far the most frequent indication for administering a paediatric pain and fever syrup, reported by 91% of parents, followed by mild to moderate pain and fever associated with cold and flu (54%) and headache (43%). Less common indications included toothache and teething ailments, migraine, and injuries. When asked about their first reaction upon noticing fever or pain in their child, the majority of parents reported ensuring the child gets adequate rest, followed by waiting to observe how symptoms develop before administering medication. Directly giving a painkiller was the third most common first response, followed by visiting the paediatrician, using a home remedy, or seeking pharmacy advice.

In terms of administration frequency, 53% of parents reported giving a children’s pain and fever syrup 2 to 3 times per year, with a mean of 3.3 occasions per year. Most parents (63%) had last administered the product within the preceding 3 months, with a mean time since last administration of 3.7 months. Regarding the flavour varieties given, strawberry was reported by 100% of respondents, reflecting the study inclusion criteria, which required use of the strawberry-flavoured product within the previous 12 months. As the use of other paediatric medications was also permitted, these data are not intended to reflect market share. Of the other flavours reported, orange was the second most frequently used, followed by raspberry, cherry, and mixed-fruit varieties.

Between 33% and 56% of children had at some point tried another (differently) flavoured suspension but finally did not prefer it. When asked about formats used in addition to syrup, drops, suppositories, and Meltlets (in the case of children aged 6–12 years) were the most frequently cited alternatives. Notably, 22% of parents reported giving pain killers exclusively in syrup form, confirming the central role of this formulation in everyday paediatric pain and fever management.

## 4. Discussion

This cross-sectional survey of 1005 German parents provides large-scale, real-world parent-reported survey data on the parental experience of administering a strawberry-flavoured ibuprofen suspension to children aged 3 months to 12 years. Among these experienced users, the findings converge on three principal observations: overall satisfaction and willingness to recommend were exceptionally high (99% and 98%, respectively); agreement with all evaluated product-specific statements exceeded 88%, with taste-related statements achieving 90% or above; and taste that the child likes ranked as the seventh most important purchase decision criterion overall—immediately after the criterion that the child does not refuse the medicine—indicating that parents rated palatability as an important consideration in their self-reported purchasing decisions rather than a peripheral one.

The prominence of taste in the purchase decision hierarchy deserves particular attention. Among the 17 product criteria evaluated, taste that the child likes (87%) ranked above strong effect (83%), dosage syringe availability (83%), and symptom relief breadth (81%), and was rated by nearly nine out of ten parents as important or very important. This finding is consistent with the established literature: Batchelor and Marriott (2014) identified palatability as one of the primary formulation design requirements for the paediatric population [3], and Elgammal et al. (2024) reported that palatability concerns were associated with clinicians deviating from first-line prescribing guidelines, illustrating that poor taste imposes systemic consequences beyond individual refusal events [8]. The present survey extends this evidence to the consumer purchase decision context, suggesting that the parents surveyed took taste into account when selecting products.

The taste-specific agreement data from Q17 and Q18 corroborate this picture at the product level. Agreement that the product has the taste most preferred by the child (88%) and ranked highest for most preferred taste (87%) were among the strongest comparative endorsements recorded. These figures are consistent with the broader European literature on strawberry-flavoured paediatric medicines. Thompson et al. (2013) found that 94% of children were willing to take a strawberry-flavoured lozenge again, versus 56% for an orange-flavoured equivalent [10], and a German hospital study reported greater acceptability of strawberry-flavoured ibuprofen compared with an orange-flavoured comparator [11]. The present survey, conducted in the home setting with 1005 parents rather than in a clinical taste-testing environment, provides an ecologically valid, real-world complement to these controlled findings at substantially greater scale, although its uncontrolled, single-product design does not permit attribution of acceptance to the strawberry flavour specifically.

The child-centred taste statements from Q18 are especially noteworthy: 90% of parents agreed that their child finds the taste pleasant and that their child likes the taste, while 88% confirmed the child takes the product without fussing or spitting out. Taken together with the comparative statement that the product is the one the child is most willing to take (83%), these results indicate high parent-reported acceptance among experienced users. Acceptability of this kind is a plausible facilitator of adherence; however, the present survey did not measure adherence, completed or missed doses, symptom resolution, or clinical outcomes, so the link between acceptability and adherence remains a hypothesis to be tested rather than a demonstrated effect. This distinction matters given the episodic nature of analgesic/antipyretic use (mean 3.3 occasions per year), in which each administration is a fresh potential refusal event. The ease-of-administration ratings (96%) are similarly parent-reported perceptions; because dosing accuracy, administration safety, syringe technique, and caregiver health literacy were not objectively assessed, these ratings should not be read as evidence of demonstrated dosing safety or of a reduction in dosing errors, even though difficulties with measuring devices are a recognised source of such errors [15].

More broadly, a systematic review of the peer-reviewed literature on palatability testing of medicines in children found considerable heterogeneity in study design, measurement scales, and reporting quality across 30 identified studies, and proposed methodological recommendations—including age-appropriate hedonic or visual analogue scales, randomised and blinded designs where feasible, and adequately powered sample sizes—for conducting reliable, age-appropriate sensory evaluations in this population [16]. The present survey’s binary agree/do not agree format and large, real-world sample complement this body of controlled taste-testing literature by adding an ecologically valid, market-scale perspective, while sharing none of its measurement granularity.

Caregivers’ role as proxy reporters of child acceptance is a well-established approach in this research field, particularly for younger children who cannot reliably self-report [17]. Blume et al. (2018) validated the Caregiver-administered Children’s Acceptance Tool (CareCAT), demonstrating strong intra- and inter-rater reliability for caregiver-reported acceptance, thereby supporting the methodological appropriateness of parental proxy assessment in real-world contexts [17]. The consistently higher agreement levels observed in the 6 to 12 year age group across both Q17 and Q18 may reflect greater verbal expressiveness of older children in communicating taste preferences to their parents, a pattern noted in the paediatric palatability literature [17]. Notably, however, children in the older group could in principle have reported taste directly; because all outcomes were nonetheless obtained from parents, they remain parent-perceived rather than child-reported.

The consistent direction of the age-related differences warrants separate consideration. Across nearly all Q17 and Q18 items, agreement was higher among parents of children aged 6–12 years than among parents of children aged 3 months to 5 years, and for several items the Wilson 95% confidence intervals of the two subgroups did not overlap, which is compatible with an age-related difference rather than with chance alone. Statistical significance and practical relevance are, however, distinct. The absolute differences amounted to 4–7 percentage points, and no minimal important difference has been established for binary claim-substantiation statements of this kind, so the practical relevance of these gaps cannot be quantified from the present data. It is equally relevant that agreement remained at or above 82% for every statement in both age groups: the age pattern therefore describes a gradient within uniformly high reported acceptability rather than an acceptability problem in younger children. Because the ceiling imposed by the binary format compresses variance at these high levels of endorsement, small absolute differences can reach statistical significance while the instrument provides no means of establishing whether they correspond to a difference that parents or children would notice in everyday use.

Several explanations may account for the consistent age pattern, none of which can be tested with the present data. First, taste perception differs developmentally, with younger children showing a more pronounced preference for sweetness and greater sensitivity to bitter stimuli, and medication-taking behaviour differs with age, in that infants and toddlers cannot yet understand why a medicine is being given and are more likely to resist administration, fuss, or spit out a dose irrespective of flavour [3,4]. Second, older children can articulate their preferences verbally, so that parents of children aged 6–12 years can base taste-related answers on what the child has actually told them, whereas parents of infants and toddlers must infer acceptance from facial expressions and behaviour [17]. Third, and closely related, the accuracy of parental proxy reporting may itself differ by child age. Because the response format offered no neutral or ‘don’t know’ option, inferential uncertainty on the part of parents of younger children is pooled with genuine disagreement, which may lower apparent agreement in the younger subgroup independently of any true difference in child acceptance. Differential accuracy of proxy reporting and a true age difference in acceptability therefore remain indistinguishable in these data. Given the number of unadjusted subgroup comparisons, these age-related findings are presented as exploratory and hypothesis-generating; testing them would require prespecified comparisons and age-appropriate direct assessment of the children themselves.

Among brand reputation criteria, paediatrician recommendation (71%) ranked second only to brand trust (73%), while pharmacy staff recommendation (59%) also reached notable levels. This underlines the continued influence of healthcare professionals on OTC purchase decisions in the paediatric analgesic category and suggests that evidence-based information on palatability and child acceptance may be relevant for communication with healthcare professionals and caregivers.

### Limitations

Several limitations warrant consideration. Given the design, several of them are central to the interpretation of the findings. First, the use of a binary agree/do not agree response format provided a straightforward measure of participant views but did not capture the intensity of agreement. The absence of a neutral or ‘don’t know’ category means that alternative interpretations of the ‘do not agree’ responses cannot be distinguished. Moreover, as the product-specific statements were positively framed, some influence of response tendencies cannot be excluded. The use of a Likert-type scale in future studies could facilitate a more granular evaluation of respondent perspectives.

Relatedly, the near-universal agreement rates are consistent with a ceiling effect, which compresses variance and constrains the interpretability of between-item and between-subgroup differences; the uniformly high endorsements should therefore be read as evidence of broad acceptability rather than as finely discriminating measurements. The market-research items are accordingly better regarded as a preliminary means of identifying candidate acceptability domains—taste, ease of administration, and willingness to take the medicine—than as validated measurements; a more rigorous, balanced instrument developed through literature review, expert and caregiver input, concept elicitation, balanced (including negatively framed) item wording, cognitive testing, and psychometric validation would strengthen future research. Pairing binary endorsement with a strength-of-agreement scale in future work would allow this gradient to be quantified directly. Second, child acceptance and taste experience were assessed via parental proxy report, which, while validated [17], may differ from direct child assessment, particularly in the younger age group; because these outcomes were obtained from parents even for children old enough to report taste themselves, they should be understood throughout as parent-perceived rather than child-reported. Third, the study was funded by the manufacturer. As with any industry-funded research, this should be considered when interpreting the findings.

Although the survey was designed and executed by an independent research agency, commercial sponsorship remains a relevant contextual factor. Respondents were not blinded to the product under evaluation, which was named explicitly in every statement; the comparative Q17 items were likewise not based on randomised or concurrent comparison—prior exposure to alternatives was heterogeneous (85% had used another brand at some unspecified time)—so those items represent subjective retrospective impressions rather than comparative-effectiveness evidence. The age-subgroup analyses, moreover, involved many comparisons without correction for multiple testing and are exploratory and hypothesis-generating: with n = 1005, differences of a few percentage points can reach significance without being practically meaningful, and should not be read as meaningful differences in acceptability. The sample was also quota-controlled for parent gender only, with no data on region, education, income, employment, household composition, child sex, or urban/rural residence, so representativeness beyond gender cannot be established and the term ‘German parents’ should be read with caution. Finally, the absence of a comparator arm limits causal attribution: the observed outcomes reflect the product as experienced holistically, and the relative contribution of strawberry flavouring versus other formulation attributes—including the provision of a dosing syringe, which was rated important by 83% of parents in this survey—cannot be disentangled. Acceptance may equally reflect sweetness, viscosity, texture, odour, mouthfeel, the dosing device, brand familiarity, or prior positive experience, none of which this design can separate from the strawberry flavour itself. A further and more fundamental limitation concerns the recruitment design. All respondents were eligible because they were aware of, and had personally administered, the strawberry-flavoured study product to a child within the preceding 12 months. However, the use of other paediatric medications during the same period was permitted and did not affect eligibility.

The satisfaction, acceptance, and taste-agreement rates reported here reflect the experiences of parents who had administered this specific product within the previous 12 months, although use of other paediatric medications during the same period was also permitted. Accordingly, the findings should be interpreted within the context of this study population and may not be fully generalisable to the wider parent population or to children without prior experience of the product.

For the same reason, the 100% strawberry-flavour exposure is an artefact of the eligibility criteria and must not be read as a market-share or head-to-head comparative signal; the figures might not indicate the proportion of all German parents or children who would prefer or accept this product relative to available alternatives. The high agreement rates may therefore be interpreted as indicating a positive level of acceptability among parents who had already used the product, rather than as an estimate of how the product might be perceived by an unselected.

These findings also have a potential, hypothesis-generating practical implication for the German reimbursement and dispensing context. Under the statutory aut-idem rule and the discount contracts (Rabattverträge) negotiated between statutory health insurers and manufacturers, pharmacies are generally obliged to substitute a prescribed medicine with the most cost-effective product containing the same active ingredient, unless the prescribing physician explicitly excludes substitution [12]. This mechanism is an established tool for cost containment. As the present survey did not assess substitution events, medication switching, adherence, comparative costs, healthcare utilisation, or clinical and economic outcomes, the findings do not permit conclusions regarding reimbursement or substitution policies. Nevertheless, they may provide an initial basis for further research exploring these questions. Moreover, it raises a testable hypothesis—that formulation acceptability might warrant consideration alongside price in paediatric substitution decisions, since a low-cost product a child accepts poorly could in principle deliver less therapeutic benefit than a marginally costlier, better-accepted alternative.

Because the present survey measured neither dose-level adherence nor downstream resource use, no cost-effectiveness estimate can be derived from these data; future research should assess whether formulation acceptability influences adherence, healthcare utilisation, and total treatment cost.

## 5. Conclusions

Among surveyed German parents with direct, recent experience of the strawberry-flavoured ibuprofen suspension, reported satisfaction (99%), willingness to recommend (98%), and perceived child acceptance were high, and agreement exceeded 90% for the taste and acceptance items. In these parents’ self-reported ratings, a taste the child likes was among the more important purchase criteria for children’s analgesic/antipyretic syrups, immediately after the criterion that the child does not refuse the medicine. Because the sample comprised exclusively established users of the product (although it did not exclude those who used other paediatric medications in the past 12 months), all acceptance and taste outcomes were parent-reported. The study was designed as a descriptive survey of user experience; therefore, the findings should be interpreted as descriptive of this study population and cannot be used to infer comparative performance, population-level acceptance, medication adherence, therapeutic efficacy, dosing safety, or cost-effectiveness.

These results add a German-market, real-world, parent-reported perspective to the literature on the potential importance of palatability for paediatric medication acceptability, a perspective that has been largely absent from published data. They are best regarded as hypothesis-generating: future research should address the present limitations through controlled comparative designs with direct child hedonic assessment, a balanced and validated instrument, and longitudinal follow-up to test whether greater acceptability translates into more complete dosing, better adherence, and improved clinical outcomes.

## Figures and Tables

**Table 1:** Sample characteristics: parent gender and age of the index child (n = 1005). The index child is the child to whom the parent had most recently given the strawberry-flavoured study product within the preceding 12 months. No further sociodemographic characteristics were collected.

Characteristic	Respondents
Parent gender—women	70%
Parent gender—men	30%
Age of index child—3 months to 3 years	28%
Age of index child—4 to 5 years	15%
Age of index child—6 to 9 years	32%
Age of index child—10 to 12 years	25%

**Table 2:** Agreement with comparative statements about Nurofen Junior Fever and Pain Suspension Strawberry (Q17; binary agree/do not agree format; values in parentheses are Wilson 95% confidence intervals; n = 1005 total; n = 428 aged 3 months–5 years; n = 577 aged 6–12 years).

Statement (Q17)	Total (n = 1005)	3 Months–5 Years (n = 428)	6–12 Years (n = 577)
…gives me fewer struggles when giving medicine to my child	93% (91–94)	92% (89–94)	93% (91–95)
…has high acceptance for my child	90% (88–92)	89% (86–92)	90% (87–92)
…has the taste that is most preferred by my child	88% (86–90)	84% (80–87)	90% (87–92)
…is the number 1 product for most preferred taste by my child	87% (85–89)	84% (80–87)	89% (86–91)
…is better accepted by my child than other medicines in syrup form	87% (85–89)	83% (79–86)	90% (87–92)
…is the medicine that my child is most willing to take	83% (81–85)	82% (78–85)	85% (82–88)

**Table 3:** Agreement with product-specific experience statements about Nurofen Junior Fever and Pain Suspension Strawberry (Q18; binary agree/do not agree format; values in parentheses are Wilson 95% confidence intervals; n = 1005 total; n = 428 aged 3 months–5 years; n = 577 aged 6–12 years). NJS = Nurofen Junior Fever and Pain Suspension Strawberry.

Statement (Q18)	Total (n = 1005)	3 Months–5 Years (n = 428)	6–12 Years (n = 577)
…is easy and safe to administer	96% (95–97)	95% (93–97)	96% (94–97)
I am satisfied with how well my child takes NJS	92% (90–94)	89% (86–92)	95% (93–96)
…is well accepted by my child	92% (90–94)	90% (87–92)	93% (91–95)
I am satisfied with the flavour of NJS	92% (90–94)	89% (86–92)	94% (92–96)
With NJS I have a good feeling that my child takes medicine without problems	91% (89–93)	88% (85–91)	93% (91–95)
My child finds the taste of NJS pleasant	90% (88–92)	88% (85–91)	92% (90–94)
My child likes the taste of NJS	90% (88–92)	86% (82–89)	93% (91–95)
My child takes NJS without problems (does not fuss or spit out)	88% (86–90)	85% (81–88)	90% (87–92)

**Table 4:** Importance of product criteria for the purchase decision of a children’s pain and fever syrup (Q19; 5-point Likert scale; values in parentheses are Wilson 95% confidence intervals; Top-2 Box: ‘important’ or ‘very important’; n = 1005).

Purchase Decision Criterion (Q19)	% Top-2 Box
Product well tolerated by child	96% (95–97)
Easy to administer	95% (94–96)
Fast-acting effect	95% (94–96)
Easy to dose	93% (91–94)
Long-lasting effect	92% (90–94)
Child does not refuse/make a fuss	91% (89–93)
Taste that child likes	87% (85–89)
Strong effect	83% (81–85)
Dosage syringe available	83% (81–85)
Relieves multiple symptoms	81% (78–83)
Does not contain artificial additives	59% (56–62)
Contains ibuprofen	48% (45–51)
Sugar-free	45% (42–48)
Allergen-free	41% (38–44)
Contains paracetamol	32% (29–35)
Does not contain paracetamol	17% (15–19)
Does not contain ibuprofen	16% (14–18)

**Table 5:** Importance of brand reputation criteria for the purchase decision of a children’s pain and fever syrup (Q20; 5-point Likert scale; values in parentheses are Wilson 95% confidence intervals; Top-2 Box: ‘important’ or ‘very important’; n = 1005).

Brand Reputation Criterion (Q20)	% Top-2 Box
From a brand I trust	73% (70–76)
Recommended by my paediatrician	71% (68–74)
From a brand that is a pain killer expert	64% (61–67)
From a well-known brand	62% (59–65)
Recommended by pharmacy staff	59% (56–62)
Inexpensive	47% (44–50)
Recommended by friends/acquaintances	42% (39–45)

## Data Availability

The datasets generated and analysed during the current study are not publicly available due to contractual obligations with the study sponsor but are available from the corresponding author on reasonable request.

## Supplementary Materials

The following supporting information can be downloaded at: https://www.mdpi.com/article/10.3390/jmahp14030056/s1. File S1: STROBE checklist (cross-sectional; adapted for a market-research survey).
