# Supplementary material for: Parent-Reported Satisfaction, Perceived Child Acceptance, and Ease of Administration of a Strawberry-Flavoured Ibuprofen Suspension for Children: A Cross-Sectional Survey of Experienced Users in Germany
Source: J Mark Access Health Policy. 2026 Sep 18;14(3):56. doi: 10.3390/jmahp14030056 (PMC13608591; doi:10.3390/jmahp14030056)
Supplement: Supplementary file 1 [file jmahp-14-00056-s001.zip › jmahp-4484294-supplementary.pdf]

Appendix — STROBE checklist (cross-sectional; adapted for a market-research survey)

Item numbers follow the STROBE statement for cross-sectional studies. “Section” refers to the revised manuscript.

#	STROBE recommendation	Addressed in
1	Title & abstract — design in title; balanced abstract summary	Title; Abstract
2	Background / rationale	Introduction
3	Objectives	Introduction (final paragraph)
4	Study design	2.1 Study Design
5	Setting, locations, dates	2.1 (Germany, May 2026)
6	Participants — eligibility criteria and sources	2.2 Study Population and Eligibility
7	Variables — outcomes and predictors	2.4 Survey Instrument; 2.5
8	Data sources / measurement (per variable)	2.4; Supplementary Questionnaire S1
9	Bias — sources and handling	2.4; 4.1 Limitations (selection, framing, proxy, ceiling)
10	Study size — how arrived at	2.3 Sample Size (p=0.5 assumption)
11	Quantitative variables — grouping	2.5 (age dichotomy; Table 1 finer bands)
12	Statistical methods	2.5 Statistical Analysis (Wilson CI; exploratory subgroup tests)
13	Participants — numbers at each stage	3.1; n=1,005 (428 / 577)
14	Descriptive data — characteristics	3.1 Sample Characteristics; Table 1
15	Outcome data	3.1–3.6; Tables 2–5
16	Main results — estimates and precision (CIs)	3.1–3.4; Tables 2–5 (Wilson 95% CIs)
17	Other analyses — subgroups	3.2–3.3 (exploratory age subgroups)
18	Key results — summary with reference to objectives	4. Discussion (opening)
19	Limitations	4.1 Limitations
20	Interpretation — cautious, considering bias	4. Discussion; 5. Conclusions
21	Generalisability / external validity	4.1 (established-user sample; gender-only quota)
22	Funding and role of funders	Funding; Conflicts of Interest
